# Supplementary material for: Community vibrancy and its relationship with safety in Philadelphia
Source: PLoS One. 2021 Dec 31;16(12):e0257530. doi: 10.1371/journal.pone.0257530 (PMC8719732; doi:10.1371/journal.pone.0257530)
Supplement: S1 File — (ZIP) [file pone.0257530.s001.zip › PONE-D-20-26848R2.final.supmaterials.pdf]

Supplementary Materials for Community vibrancy and its relationship with safety in Philadelphia

Wichinpong Park Sinchaisri and Shane T. Jensen

1 Spatial Distribution of Community Measures in Philadelphia

In Fig S1 (left), we show the total number of block party events within each neighborhood of Philadelphia, aggregated across the entire time span of our data (2006-2016). We see in Fig S1 (left) that neighborhoods that have the largest total number of block party events are in the North Philadelphia area. West Philadelphia and South Philadelphia also have several neighborhoods with a large total number of block party events, whereas the outlying suburban communities in the Northwest and Northeast parts of the city have relatively few total number of block party events.

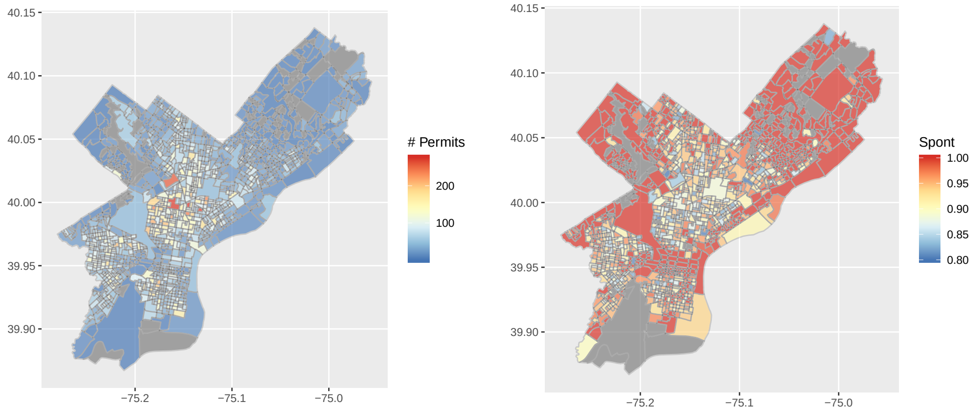

Fig S1. Left: Map of Philadelphia showing the total number of permits per neighborhood. **Right:** Map of Philadelphia showing the proportion of spontaneous to regular events per neighborhood

In Fig S1 (right), we show the spontaneous within each neighborhood of Philadelphia, based on the total number of spontaneous and regular events across the entire time span of our data (2006-2016). It is interesting to observe that while North Philadelphia contains the neighborhoods with the largest total number of block party events, these North Philadelphia neighborhoods also have a lower spontaneous proportion than the areas of the city that have a smaller number of block party events. Center city and the Northwest and Northeast suburban communities contain the neighborhoods with the highest spontaneous proportions in Philadelphia.

2 Monthly Trends in Community Measures in Philadelphia

In Fig S2, we show the variation by month of the total number of block party events and the spontaneous proportion of block party events that we introduced in our main paper. Note that 2016 is not included in Fig S2 since we only have data for part of that year.

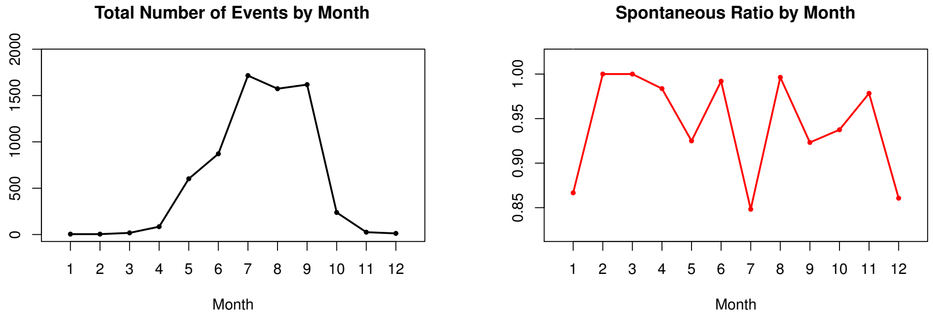

Fig S2. Monthly trends in the total number of block party events and the spontaneous proportion of block party events.

In terms of monthly or seasonal variation, we see a clear trend in the left of Fig S2 for a greater number of block party events during the warmer months from May (5) to September (9). The spontaneous proportion is lower in May (5), July (7) and September (9) which is due to the prominence of regular holidays (Memorial day, 4th of July, and Labor day) during those months.

3 Distribution of Total Crimes across Philadelphia Neighborhoods

Fig S3 is a map of the spatial distribution of total crimes per year (averaged over the years from 2006-2015) in Philadelphia, as well as the log transformation of total crimes per year.

Fig S4 (left) gives the distribution of total crimes over the entire time span across these 1336 neighborhoods. Since that distribution is highly skewed, we will focus on the log transformation of crime in our analyses which has the more symmetric distribution shown in Fig S4 (right).

4 Correlations between Crime, Community Vibrancy and Other Neighborhood Characteristics

Fig S5 provides correlations between our measures of community vibrancy, several crime measures and the demographic, economic and built environment measures collected for Philadelphia.

We observe that spontaneous proportion of block parties is not strongly correlated with any of these other neighborhood characteristics. However, the total number of block party permits is correlated with both economic measures (median income and poverty index) as well as the proportion of black residents in a neighborhood. We also

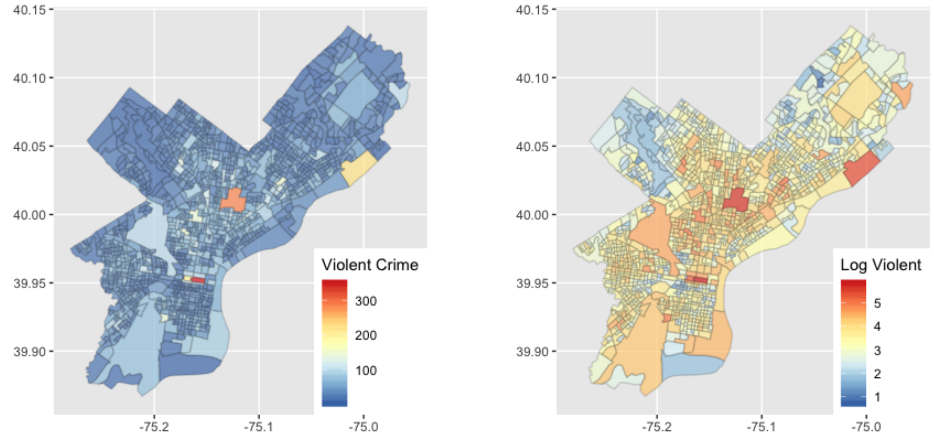

Fig S3. Distribution of violent crime over the block groups of Philadelphia. **Left:** violent crimes per block group, averaged over the years from 2006 to 2015. **Right:** logarithm of violent crimes per block group, averaged over the years from 2006 to 2015.

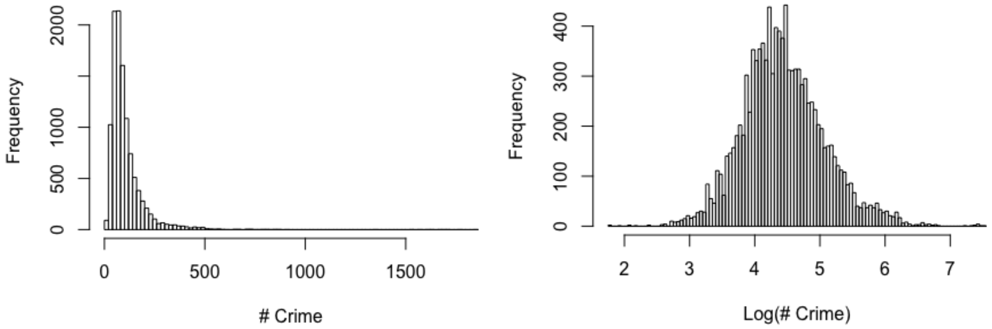

Fig S4. Left: Distribution of the total number of crimes by US census block group in Philadelphia and Right: Distribution of the logarithm of total crimes.

see that the total number of block party permits is correlated with our measures of crime incidence, and that those crime measures are strongly correlated with several other neighborhood characteristics.

5 Linear Regression Analysis of Total Crime and Community Vibrancy

In our main paper, we discuss the results from four different regressions that represent each combination of our two community vibrancy measures and our two regression model specifications,

1. Ordinary least squares (OLS) regression of the logarithm of total crime incidence $\log(y_i)$ on the number of total events C_i and other neighborhood characteristics $\mathbf{X}_i$

2. Ordinary least squares (OLS) regression of the logarithm of total crime incidence $\log(y_i)$ on the spontaneous proportion C_i and other neighborhood characteristics $\mathbf{X}_i$

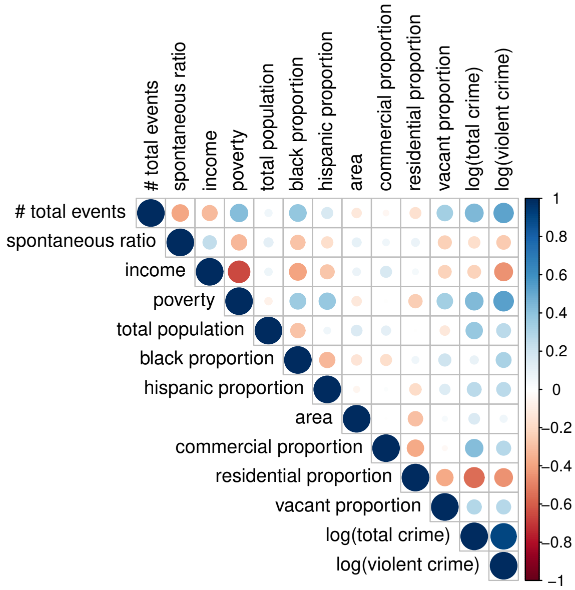

Fig S5. Correlations between community vibrancy, demographic, economic, land use and crime measures across all block groups in Philadelphia. Blue indicates a positive correlation, while red reflects a negative correlation. The darker the shade, the larger the magnitude of the correlation.

3. Negative binomial regression of total crime incidence $\log(y_i)$ on the number of total events C_i and other neighborhood characteristics $\mathbf{X}_i$
4. Negative binomial regression of total crime incidence $\log(y_i)$ on the spontaneous proportion C_i and other neighborhood characteristics $\mathbf{X}_i$

Table S1 displays the parameter estimates and model fit statistics for the four regression models outlined above.

We see in Table S1 that OLS regression models are a better fit to the data than the negative binomial regression models in terms of root mean square error (RMSE). We also see that the number of total permits is significantly positively associated with total crimes (models 1 and 3), whereas the spontaneous proportion is non-significantly negatively associated with total crimes (models 2 and 4).

We also see that most neighborhood characteristics have significant partial effects, which suggests that each of these economic, demographic and land use characteristics have an association with crime, even after accounting for the other characteristics included in the model. Higher levels of poverty and larger commercial proportions are associated with higher levels of total crime in each of the four models, whereas higher proportions of park space and residential land use are associated with lower levels of total crime.

5.1 Regression Analysis of Trends over Time

In our main paper, we used regression models to explore the neighborhoods factors that are predictive of whether or not a neighborhood has a significant trend over time in either crime or our measures of community vibrancy. Specifically, we fit the four different logistic regression models enumerated below:

1. Logistic regression with significantly increasing trend in community (or not) as the outcome and neighborhood characteristics $\mathbf{X}_i$ (including indicators of trends

Table S1. Regression model summaries for four different models with the number of total crimes as the outcome variable

	Dependent variable:			
	Log number of total crimes		Number of total crimes	
	OLS		*negative binomial*	
	(1)	(2)	(3)	(4)
# Permits	0.002*** (0.0003)		0.002*** (0.0003)	
Spontaneity ratio		−0.281$^+$ (0.158)		−0.229 (0.156)
Log income	0.014 (0.030)	0.016 (0.031)	0.026 (0.030)	0.028 (0.031)
Poverty	0.681*** (0.090)	0.751*** (0.093)	0.733*** (0.089)	0.796*** (0.092)
Log population	0.633*** (0.027)	0.713*** (0.027)	0.629*** (0.027)	0.708*** (0.026)
Black	0.310*** (0.037)	0.430*** (0.036)	0.296*** (0.037)	0.411*** (0.036)
Hispanic	0.399*** (0.070)	0.498*** (0.072)	0.407*** (0.069)	0.493*** (0.071)
Area (10^6)	0.177*** (0.023)	0.162*** (0.024)	0.203*** (0.023)	0.193*** (0.024)
Commercial	2.700*** (0.172)	2.532*** (0.177)	2.739*** (0.171)	2.553*** (0.175)
Residential	−1.236*** (0.103)	−1.437*** (0.105)	−1.357*** (0.102)	−1.587*** (0.103)
Vacant	0.282 (0.208)	0.479* (0.214)	0.194 (0.206)	0.375$^+$ (0.212)
Transportation	−0.024** (0.009)	−0.030** (0.010)	−0.023* (0.009)	−0.029** (0.009)
Industrial	−0.017 (0.160)	−0.224 (0.164)	−0.072 (0.159)	−0.322* (0.162)
Park	−0.819*** (0.165)	−0.995*** (0.169)	−0.853*** (0.164)	−1.065*** (0.167)
Civic	0.293* (0.137)	0.107 (0.141)	0.292* (0.136)	0.097 (0.139)
Constant	2.714*** (0.384)	2.561*** (0.427)	2.725*** (0.382)	2.537*** (0.422)
Observations	1,265	1,265	1,265	1,265
R^2	0.718	0.697		
Adjusted R^2	0.715	0.694		
Akaike Inf. Crit.			19,686.970	19,765.860
RMSE	0.3198	0.3313	0.3403	0.3488
Note:				+p<0.01; * p <0.05; **p<0.01; ***p<0.001

in crime) as the predictors

2. Logistic regression with significantly decreasing trend in community (or not) as the outcome and neighborhood characteristics $\mathbf{X}_i$ (including indicators of trends in crime) as the predictors

3. Logistic regression with significantly increasing trend in crime (or not) as the outcome and neighborhood characteristics $\mathbf{X}_i$ (including indicators of trends in community) as the predictors

4. Logistic regression with significantly decreasing trend in crime (or not) as the outcome and neighborhood characteristics $\mathbf{X}_i$ (including indicators of trends in community) as the predictors

Table S2 displays the parameter estimates and model fit statistics for the four logistic regression models listed above, where we use the number of block party permits as our measure of community. We see that log income is a strong predictor of significantly increasing trends in block party permits (model 1) and that vacant proportion is a strong predictor of significantly decreasing trends in block party permits (model 2). We also see that industrial land use is a strong predictor of a significantly increasing trend in crime (model 3) and that the Hispanic proportion is a strong predictor of a significantly decreasing trend in crime (model 4). It is worth noting that for predicting significantly increasing trends in either block party permits or crimes, there are so few cases of either of those two outcomes ($n = 94$ in model 1 and $n = 38$ in model 3) which which gives us limited power to detect strong associations.

Table S3 displays the parameter estimates and model fit statistics for another four logistic regression models in which we use the spontaneity proportion as our measure of

Table S2. Logistic regression model results for predicting neighborhoods with different types of significant trends over time

	Dependent variable:			
	Permits +	Permits −	Crimes +	Crimes −
	(1)	(2)	(3)	(4)
Log income	0.087^{***} (0.024)	0.001 (0.032)	−0.010 (0.016)	0.110^{*} (0.046)
Poverty	0.002 (0.071)	0.148 (0.097)	0.034 (0.047)	0.034 (0.137)
Log population	0.015 (0.021)	0.033 (0.028)	0.002 (0.014)	0.063 (0.040)
Black	−0.024 (0.027)	0.018 (0.037)	0.036^{*} (0.018)	−0.058 (0.052)
Hispanic	0.019 (0.055)	0.100 (0.074)	0.001 (0.036)	0.328^{**} (0.105)
Area (10^6)	−0.013 (0.018)	−0.010 (0.025)	0.006 (0.012)	−0.040 (0.035)
Commercial	−0.013 (0.136)	−0.215 (0.186)	0.222^{*} (0.089)	−0.224 (0.261)
Residential	−0.110 (0.080)	0.111 (0.109)	0.022 (0.053)	-0.271^{+} (0.154)
Vacant	−0.242 (0.164)	0.723^{**} (0.223)	0.137 (0.108)	0.521^{+} (0.316)
Transportation	−0.003 (0.007)	−0.006 (0.010)	0.010^{*} (0.005)	−0.009 (0.014)
Industrial	−0.165 (0.126)	−0.067 (0.172)	0.256^{**} (0.083)	−0.109 (0.242)
Park	0.024 (0.130)	−0.030 (0.177)	−0.029 (0.085)	−0.072 (0.250)
Civic	0.179^{+} (0.108)	0.109 (0.147)	−0.038 (0.071)	0.127 (0.208)
Crimes +	0.024 (0.044)	−0.053 (0.059)		
Crimes −	0.003 (0.015)	0.012 (0.020)		
Permits +			0.008 (0.019)	0.012 (0.055)
Permits −			−0.013 (0.014)	0.031 (0.040)
Constant	-0.818^{**} (0.304)	−0.230 (0.413)	0.048 (0.200)	-0.970^{+} (0.584)
Outcome = 1	94	184	38	589
Observations	1,265	1,265	1,265	1,265
Log Likelihood	−62.720	−452.367	468.369	−888.767
Akaike Inf. Crit.	157.439	936.734	−904.738	1,809.533
Note:				+p<0.01; * p <0.05; **p<0.01; ***p<0.001

community. We find that several factors are significantly associated with a positive trend over time in spontaneity proportion, e.g. poverty, population size, proportions of Black and Hispanic residents, as well as residential and vacant land use (column 1). On the other hand, the only significant predictors of the much smaller set of neighborhoods with a negative trend in spontaneous proportion are the proportion of Black residents and residential and civic land uses (column 2). There is no significant association between trends over time in crime and trends over time in the spontaneous proportion

Table S3. Logistic regression model results for predicting neighborhoods with different types of significant trends over time

	Dependent variable:			
	Spont +	Spont −	Crimes +	Crimes −
	(1)	(2)	(3)	(4)
Log income	0.041 (0.037)	0.016 (0.013)	−0.009 (0.016)	0.109^{*} (0.046)
Poverty	0.312^{**} (0.112)	0.001 (0.040)	0.033 (0.047)	0.024 (0.137)
Log population	0.085^{**} (0.032)	0.009 (0.011)	0.002 (0.014)	0.060 (0.040)
Black	0.289^{***} (0.043)	-0.041^{**} (0.015)	0.036^{*} (0.018)	−0.069 (0.053)
Hispanic	0.347^{***} (0.086)	−0.026 (0.030)	0.001 (0.036)	0.317^{**} (0.105)
Area (10^6)	-0.055^{+} (0.029)	0.010 (0.010)	0.006 (0.012)	−0.038 (0.035)
Commercial	-0.448^{*} (0.214)	0.023 (0.076)	0.224^{*} (0.090)	−0.212 (0.262)
Residential	-0.335^{**} (0.126)	0.168^{***} (0.045)	0.020 (0.053)	-0.260^{+} (0.155)
Vacant	0.687^{**} (0.258)	0.211^{*} (0.092)	0.128 (0.108)	0.501 (0.316)
Transportation	0.005 (0.011)	0.002 (0.004)	0.010^{*} (0.005)	−0.010 (0.014)
Industrial	−0.281 (0.198)	0.113 (0.071)	0.255^{**} (0.083)	−0.104 (0.242)
Park	−0.052 (0.204)	0.088 (0.073)	−0.029 (0.085)	−0.073 (0.250)
Civic	−0.191 (0.169)	0.211^{***} (0.060)	−0.038 (0.071)	0.133 (0.208)
Crimes +	0.001 (0.068)	−0.001 (0.024)		
Crimes −	0.030 (0.023)	0.003 (0.008)		
Spont +			−0.002 (0.012)	0.045 (0.035)
Spont −			−0.003 (0.033)	0.037 (0.097)
Constant	-0.877^{+} (0.476)	−0.279 (0.169)	0.041 (0.200)	−0.936 (0.584)
Outcome = 1	313	27	38	589
Observations	1,265	1,265	1,265	1,265
Log Likelihood	−632.752	675.492	467.787	−888.171
Akaike Inf. Crit.	1,297.503	−1,318.984	−903.574	1,808.342

Note: +p<0.01; * p <0.05; **p<0.01; ***p<0.001

6 Additional Aggregate Regression Models

In our main paper, we compare the results from four different regressions that represent each combination of our two community vibrancy measures and our two regression model specifications,

1. Ordinary least squares (OLS) regression of the logarithm of total crime incidence $\log(y_i)$ on the number of total events C_i and other neighborhood characteristics $\mathbf{X}_i$
2. Ordinary least squares (OLS) regression of the logarithm of total crime incidence $\log(y_i)$ on the spontaneous proportion C_i and other neighborhood characteristics $\mathbf{X}_i$
3. Negative binomial regression of total crime incidence $\log(y_i)$ on the number of total events C_i and other neighborhood characteristics $\mathbf{X}_i$
4. Negative binomial regression of total crime incidence $\log(y_i)$ on the spontaneous proportion C_i and other neighborhood characteristics $\mathbf{X}_i$

In this section, we provide results from a similar set of regressions but with (a) just violent crimes, (b) just non-violent crimes or (c) just vice crimes as the outcome variable. Tables S4, S5, and S6 displays the parameter estimates and model fit statistics for the four regression models listed above but with violent crime, non-violent crime and vice crime as the outcome variable, respectively.

We generally observe similar results in Tables S4-S6 to Table S1 in our main paper where total crimes was the outcome variable. The partial effects for most neighborhood characteristics are significant, suggesting that each of these economic, demographic and land use characteristics are associated with violent, non-violent and vice crime. Similar to the models for total crime in our main paper, we see that the number of total permits is significantly positively associated with violent, non-violent and vice crimes, whereas the spontaneous proportion is negatively associated with violent, non-violent and vice crimes (though only significant for vice crimes).

Table S4. Results from linear regression models with the number of violent crimes as the outcome variable

	Dependent variable:			
	Log number of violent crimes		Number of Violent Crimes	
	OLS		*negative binomial*	
	(1)	(2)	(3)	(4)
# Permits	0.003*** (0.0003)		0.002*** (0.0003)	
Spontaneous ratio		−0.364* (0.180)		−0.267 (0.176)
Log income	−0.190*** (0.034)	−0.188*** (0.035)	−0.189*** (0.033)	−0.191*** (0.034)
Poverty	0.431*** (0.103)	0.504*** (0.106)	0.525*** (0.101)	0.585*** (0.103)
Log population	0.626*** (0.031)	0.711*** (0.030)	0.615*** (0.030)	0.697*** (0.030)
Black	0.683*** (0.042)	0.809*** (0.041)	0.626*** (0.041)	0.741*** (0.040)
Hispanic	0.625*** (0.079)	0.727*** (0.082)	0.559*** (0.078)	0.638*** (0.080)
Area (10^6)	0.142*** (0.026)	0.126*** (0.027)	0.195*** (0.026)	0.180*** (0.026)
Commercial	2.262*** (0.197)	2.083*** (0.202)	2.233*** (0.192)	2.028*** (0.196)
Residential	−1.145*** (0.118)	−1.360*** (0.119)	−1.198*** (0.116)	−1.451*** (0.116)
Vacant	−0.270 (0.237)	−0.065 (0.244)	−0.435+ (0.232)	−0.282 (0.238)
Transportation	−0.062*** (0.011)	−0.068*** (0.011)	−0.058*** (0.010)	−0.064*** (0.011)
Industrial	−0.140 (0.183)	−0.361+ (0.187)	−0.213 (0.179)	−0.480** (0.182)
Park	−0.838*** (0.188)	−1.026*** (0.193)	−0.841*** (0.185)	−1.064*** (0.189)
Civic	0.491** (0.157)	0.291+ (0.160)	0.698*** (0.153)	0.481** (0.156)
Constant	2.811*** (0.439)	2.709*** (0.485)	2.967*** (0.431)	2.865*** (0.474)
Observations	1,265	1,265	1,265	1,265
R^2	0.699	0.680		
Adjusted R^2	0.696	0.677		
Akaike Inf. Crit.			15,311.680	15,378.530
RMSE	0.3653	0.3767	0.3863	0.3937
Note:	+p<0.01; * p <0.05; **p<0.01; ***p<0.001			

Table S5. Results from linear regression models with the number of non-violent crimes as the outcome variable

	Dependent variable:			
	Log number of non-violent crimes		Number of non-violent crimes	
	OLS		*negative binomial*	
	(1)	(2)	(3)	(4)
# Permits	0.002*** (0.0002)		0.001*** (0.0002)	
Spontaneous ratio		−0.187 (0.151)		−0.127 (0.151)
Log income	0.085** (0.029)	0.086** (0.030)	0.097*** (0.029)	0.099*** (0.029)
Poverty	0.579*** (0.088)	0.622*** (0.089)	0.609*** (0.088)	0.649*** (0.089)
Log population	0.695*** (0.027)	0.744*** (0.026)	0.691*** (0.026)	0.738*** (0.026)
Black	0.044 (0.036)	0.118*** (0.035)	0.023 (0.036)	0.092** (0.035)
Hispanic	0.054 (0.068)	0.115+ (0.069)	0.020 (0.068)	0.072 (0.068)
Area (10^6)	0.166*** (0.023)	0.157*** (0.023)	0.193*** (0.022)	0.188*** (0.023)
Commercial	2.839*** (0.168)	2.735*** (0.170)	2.913*** (0.167)	2.803*** (0.168)
Residential	−1.282*** (0.101)	−1.406*** (0.100)	−1.415*** (0.100)	−1.550*** (0.099)
Vacant	0.238 (0.203)	0.359+ (0.205)	0.197 (0.202)	0.308 (0.204)
Transportation	0.001 (0.009)	−0.003 (0.009)	0.002 (0.009)	−0.001 (0.009)
Industrial	0.258+ (0.156)	0.130 (0.157)	0.227 (0.156)	0.080 (0.156)
Park	−0.732*** (0.161)	−0.841*** (0.162)	−0.776*** (0.161)	−0.906*** (0.161)
Civic	0.148 (0.134)	0.032 (0.135)	0.162 (0.133)	0.050 (0.134)
Constant	0.606 (0.376)	0.523 (0.408)	0.620+ (0.375)	0.487 (0.406)
Observations	1,265	1,265	1,265	1,265
R^2	0.705	0.696		
Adjusted R^2	0.701	0.693		
Akaike Inf. Crit.			16,509.560	16,540.180
RMSE	0.3127	0.3172	0.3379	0.3413
Note:			+p<0.01; * p <0.05; **p<0.01; ***p<0.001	

Table S6. Results from linear regression models with the number of vice crimes as the outcome variable

	Dependent variable:			
	Log number of vice crimes		Number of vice crimes	
	OLS		*negative binomial*	
	(1)	(2)	(3)	(4)
# Permits	0.008*** (0.001)		0.007*** (0.001)	
Spontaneous ratio		−1.461** (0.538)		−0.991* (0.387)
Log income	−0.343*** (0.102)	−0.339** (0.105)	−0.164* (0.073)	-0.144^{+} (0.076)
Poverty	1.023*** (0.308)	1.226*** (0.316)	1.510*** (0.220)	1.638*** (0.228)
Log population	0.403*** (0.093)	0.659*** (0.091)	0.332*** (0.066)	0.599*** (0.066)
Black	1.716*** (0.126)	2.073*** (0.123)	1.391*** (0.091)	1.721*** (0.089)
Hispanic	1.695*** (0.238)	1.969*** (0.244)	1.924*** (0.169)	2.157*** (0.175)
Area (10^6)	0.031 (0.079)	−0.013 (0.081)	0.140* (0.056)	0.065 (0.058)
Commercial	2.866*** (0.588)	2.344*** (0.603)	2.352*** (0.418)	1.749*** (0.432)
Residential	−2.804*** (0.352)	−3.439*** (0.355)	−2.268*** (0.253)	−3.037*** (0.257)
Vacant	−0.439 (0.709)	0.125 (0.729)	−0.074 (0.504)	0.365 (0.521)
Transportation	−0.074* (0.031)	−0.095** (0.032)	−0.095*** (0.022)	−0.116*** (0.023)
Industrial	−1.721** (0.547)	−2.376*** (0.558)	−1.395*** (0.389)	−2.274*** (0.401)
Park	−1.453** (0.563)	−2.006*** (0.576)	−1.301** (0.404)	−1.835*** (0.416)
Civic	−0.197 (0.469)	-0.789^{+} (0.478)	−0.009 (0.334)	-0.643^{+} (0.343)
Constant	3.751** (1.313)	3.826** (1.449)	2.622** (0.942)	2.116* (1.047)
Observations	1,265	1,265	1,265	1,265
R^2	0.554	0.528		
Adjusted R^2	0.549	0.522		
Akaike Inf. Crit.			12,966.920	13,078.290
RMSE	1.0932	1.1257	0.9551	0.9951
Note:			+p<0.01; * p <0.05; **p<0.01; ***p<0.001	
